# Description de *Tunga bonneti* n. sp. du Chili (Siphonaptera : Tungidae) et notes sur sa spécificité, sa chorologie, son dermecos et sa phénologie

**DOI:** 10.1051/parasite/2012193207

**Published:** 2012-08-15

**Authors:** J.-C. Beaucournu, T. Mergey, S. Muñoz-Leal, D. González-Acuña

**Affiliations:** 1 Laboratoire de Parasitologie et Zoologie appliquée, Faculté de Médecine 2, avenue du Professeur Léon Bernard 35043 Rennes Cedex France , et Institut de Parasitologie de l’Ouest, même adresse; 2 Laboratoire de Parasitologie, Centre Hospitalo-Universitaire de Pontchaillou 2, rue Henri le Guilloux 35000 Rennes; 3 Facultad de Ciencias Veterinarias, Universidad de Concepción, Av. Vicente Méndez 595 Chillán Chili

**Keywords:** *Tunga bonneti* n. sp., *Tunga libis*, description, hôte, chorologie, phénologie, *Tunga bonneti* n. sp., *Tunga libis*, description, host, chorology, phenology

## Abstract

*Tunga libis* a été à tort signalée du Chili par Smit (1968). Il s’agit en fait d’une espèce nouvelle, relativement peu rare, que nous décrivons ici. Non seulement les individus libres sont connus dans les deux sexes, mais également les femelles enkystées ou néosomiques. Des données sont apportées sur divers aspects de sa biologie. Nous pensons que ces apports seront utilisables pour affiner nos connaissances sur les autres *Tunga*, en particulier la capture des mâles, toujours rares, voire inconnus.

## Abstract (en)

Smit (1968) misreported *Tunga libis* from Chile. Here we describe this flea as a new species. We analyzed free life females, male and embedded females. Moreover, we include data about its natural history and biology. These data will allow a better understanding about other *Tunga* fleas, and particularly for collecting males, which remain unknown to date.

## Abstract (es)

*Tunga libis* fue reportada erróneamente para Chile por Smit (1968). En este trabajo describimos a esta pulga como una nueva especie, en base a la descripción de uno macho y hembras de vita libre y hembras enquistadas o neosómicos. Además, se aportan datos sobre diversos aspectos su biología. Esta información permitirá una mejor comprensión sobre los otros representantes del género *Tunga*, en particular, sobre la colecta de los machos que, hasta la fecho, permanecen desconocidos.

## Introduction

Smit (1962) a décrit la puce-chique *Tunga libis* sur un exemplaire unique (mâle holotype) de l’Équateur où il fut collecté sur *Akodon mollis* Thomas, 1894 (Rodentia – Muridae – Sigmodontinae) à Riobamba (1° 40’ 10’’ S – 78° 39’ O, 2 200 m). En 1968, cet auteur estimait avoir identifié cette même espèce au Chili et décrivit le sexe femelle sur deux exemplaires libres, ce qui est assez rare dans le genre *Tunga*. Il désigne l’un d’eux comme néallotype : Dunas de las Cruces, province de Santiago (environ 50 km au sud de Valparaiso), entre le 13 octobre et le 25 novembre 1959. Ces exemplaires furent trouvés dans un piège Barber, piège à insectes contenant du formaldéhyde.

Nous avions identifié à cette espèce deux exemplaires (un mâle et une femelle non fécondée) collectés au Chili, sur *Phyllotis darwini* (Waterhouse, 1837) (Rodentia – Muridae – Sigmodontinae) dans deux localités suivies pour la faune ectoparasitaire des Rongeurs. Ceci nous a amenés à revoir le matériel antérieur de ces mêmes localités, matériel non identifié comme appartenant à des Siphonaptères. À notre satisfaction, quelques tubes considérés comme renfermant des larves de *Cuterebra* (Diptera – Cuterebridae), mouche myiasigène, contenaient en fait des femelles enkystées, ou néosomiques, appartenant au genre *Tunga* ; ces femelles provenaient de la même espèce-hôte, *Phyllotis darwini*, que les individus cidessus. Diverses photographies prises à cette époque montrèrent *a posteriori* d’autres femelles néosomiques *in situ*. Des recherches de terrain récentes axées sur cette *Tunga* nous ont montré qu’elle était présente en plusieurs autres points du pays. Grâce à l’amabilité de Mlle T. Howard du British Museum (Natural History), l’holotype de l’Équateur et les deux femelles du Chili ont pu être examinés et il est indiscutable que nous avons deux espèces bien distinctes, *T. libis* Smit, 1962, représentée seulement par le mâle holotype, et un autre taxon, *Tunga bonneti* n. sp. que nous décrivons ici et qui rassemble notre matériel, mais aussi les deux femelles étudiées par Smit (1968), dont le pseudoallotype de *libis*. Cette nouvelle espèce est, pour le moment, seulement connue du Chili où, dans des biotopes précis, elle semble relativement peu rare. Le fait que dans le genre *Tunga* les femelles parasites soient entièrement enchâssées ou enkystées dans les tissus de l’hôte ne permet pas de préjuger de la nature de ce parasite pour un collecteur non spécialisé, et explique parfaitement cette méconnaissance. Rappelons que le mâle de *Tunga caecata* (Enderlein, 1901), espèce décrite du Brésil, n’est toujours pas connu.

Des problèmes taxonomiques demeurent cependant. Barnes & Radovsky (1969) ont décrit du nord du Mexique une espèce nouvelle de *Tunga*, *T. monositus*, inféodée comme *T. libis* et *T. bonneti* aux rongeurs Sigmodontinae. Le nouveau taxon de Barnes & Radovsky ne peut être confondu avec *T. libis*, mais ces auteurs écrivent (page 19, 2ème colonne) : “*We have examined additional* Tunga *material representing undescribed species from Peru that are also found on cricetids (*Phyllotis*)*”, et dans les Remerciements (page 35, 2ème colonne) : “*We are most grateful... to Dr Robert Traub... who examined our material and indicated its distinctness from an undescribed* Tunga *that he is studying*”. Il y a, nous semble-t-il, une petite contradiction entre ces deux citations extraites du même article, mais l’important est de noter que, de façon presque contemporaine, *T. libis* – ses pseudo-femelles du Chili – et *T. monositus* ont été décrits. En toute logique, *T. libis* étant connu de l’Équateur, mais non du Chili où existe la troisième espèce que nous décrivons ; il semble évident que le Pérou, inséré entre ces deux pays, possède au moins l’une de ces deux puces dans sa faune, même si la capture n’en a pas encore été officialisée. La description de cette *Tunga* du Pérou, étudiée par Traub, n’a en effet jamais été publiée. Le Dr J. Rawlins, curateur du Carnegie Museum National History, où sont déposés les Collections R. Traub, a bien voulu sur notre demande rechercher cette “*undescribed species*”, mais sans succès. Nous pouvons suggérer, comme simple hypothèse, que Traub ayant eu connaissance de la description de *T. libis*, et/ou de celle des femelles chiliennes qui, nous l’avons dit, ne concernent pas *T. libis*, a constaté une ressemblance dans l’un ou l’autre sexe avec l’espèce qu’il étudiait et en a différé l’étude *sine die*. Le point positif de ceci était qu’il y avait au Pérou une *Tunga* en attente d’identification, *libis* ou *bonneti*. Or, récemment Whiting *et al.* (2008) signalent de ce pays, dans une liste d’espèces de Siphonaptères étudiés sur un plan moléculaire, *T. libis* sur *Phyllotis andium*. Hastriter, l’un des co-auteurs de cet article, nous a écrit que cette identification fut faite dans l’ignorance de l’existence de *T. bonneti* et qu’elle était douteuse. Des photographies adressées par ce collègue nous permettent toutefois de confirmer la diagnose de *T. libis* au Pérou.

Dans le même ordre d’idées, Linardi & Guimarães (Sifonápteros do Brasil, 2000) citent un travail de Linardi & Botelho (1985) qui ont collecté au Brésil des femelles néosomiques de *Tunga* sp. sur *Oryzomys nigripes* et *Nectomys squamipes* (Rodentia – Sigmodontinae). Ces auteurs estimaient qu’il s’agissait de deux espèces dont l’une pouvait être *T. monositus*, mais Linardi (*in litt*., 30 octobre 2011) nous a fait remarquer que cette détermination devait être considérée comme inexacte. Les photographies publiées (*in* Linardi & Guimarães, *op. cit.*, figures 62 et 63) écartent par ailleurs toute parenté avec *T. bonneti* n. sp. ; pour ce qui est de *T. libis*, elle peut être écartée par le développement de l’oeil qui est différent de celui de l’espèce brésilienne. Par ailleurs, une nouvelle espèce, *Tunga bossii* Avelar, Linharès & Linardi (2012), du Brésil, pourrait correspondre à l’un des néosomes signalés par Linardi & Guimarães (*op. cit.*).

## Résultats

### *Tunga bonneti* n. sp. Beaucournu & González-Acuña

Synonymie : *Tunga libis* Smit, 1962, *in* Smit, 1968, les deux femelles de “Dunas de las Cruces, Santiago”, Chili, *err. det*.

Matériel de description : ♂ holotype sur *Phyllotis darwini* (Waterhouse, 1837), Quebrada Higuera (Parque Nacional Llanos de Challe), province de Huasco, 14 décembre 2010 ; ♀ allotype (ex ♀ néallotype de *T. libis*), libre (piège Barber), Dunas de las Cruces, province de Santiago, entre le 13 octobre et le 25 novembre 1959 ; ♀ paratype (ex ♀ paratype de *T. libis*) libre (piège Barber), Dunas de las Cruces, province de Santiago, entre le 13 octobre et le 25 novembre 1959 ; ♀ paratype, non enkystée, sur *Phyllotis darwini*, Parque Nacional Llanos de Challe, province de Huasco, 13 décembre 2010 (cet exemplaire a été depuis détruit accidentellement) ; ♀ paratype, enkystée (ou néosomique) près de la base de la queue, même hôte, Reserva Nacional Pan de Azúcar, province de Chañaral, 4 novembre 2010 ; ♀ paratype, enkystée dans la partie médiane du pavillon de l’oreille, même hôte, Reserva Nacional Las Chinchillas, province de Choapa, 22 mars 2011 ; ♀ paratype, enkystée près de la base de la queue sur *Phyllotis xanthopygus* (Waterhouse, 1837), Quebrada del Inca (Ollagüe) province de El Loa, 13 octobre 2011 ; deux ♀ paratypes, enkystées près de la base de la queue sur deux *Phyllotis xanthopygus*, Tal-Tal, province de Antofagasta, 31 octobre 2011.

Le néosome (c’est-à-dire l’abdomen à hyper-développement insolite, développement lié ici au parasitisme) nous est donc connu. Nous avons pu en étudier cinq exemplaires (sur 68 notés). Ils ont été extraits de l’organe parasité, queue ou pavillon de l’oreille, puis, sous contrôle d’une loupe binoculaire, fendus et éclaircis. Le côté gauche de l’insecte a parfois été enlevé de façon à permettre à la capsule céphalique d’être bien dégagée ; la persistance de la paroi droite du corps peut générer un certain degré d’obliquité par rapport au plan optique et entraîner des artefacts à l’observation, et ce, particulièrement lors des dessins. Nous avons toutefois mis à profit cette incision pour repérer et prélever la spermathèque qui a éventuellement été montée à part : celle-ci est toujours, chez les *Tunga* que nous avons étudiées (*penetrans*, *caecata*, *monositus*, *trimamillata* et *bonneti*), située en haut et un peu en avant de la plaque anale (obs. pers.), à droite évidemment.

*Derivatio nominis* : cette espèce est dédiée au Médecin de première classe Gustave Bonnet qui a publié en 1867 une remarquable étude anatomique, écologique et médicale... sur *Pulex penetrans*, la puce pénétrante, ou l’une des deux puces-chiques pouvant parasiter l’homme.

Dépôt des types : holotype, trois néosomes paratypes et la femelle libre paratype de *T. libis* (considérée ici comme paratype de *T. bonneti*) sont dans les collections du premier auteur (ultérieurement déposées au Laboratoire d’Entomologie du Muséum National d’Histoire Naturelle, Paris) ; l’allotype et un néosome sont déposés au British Museum (Natural History) ; les autres néosomes sont au Laboratoire de Parasitologie de la Facultad de Ciencias Veterinarias, Universidad de Concepción, Chillán, Chili.

### Description

**Figures 1–12.**
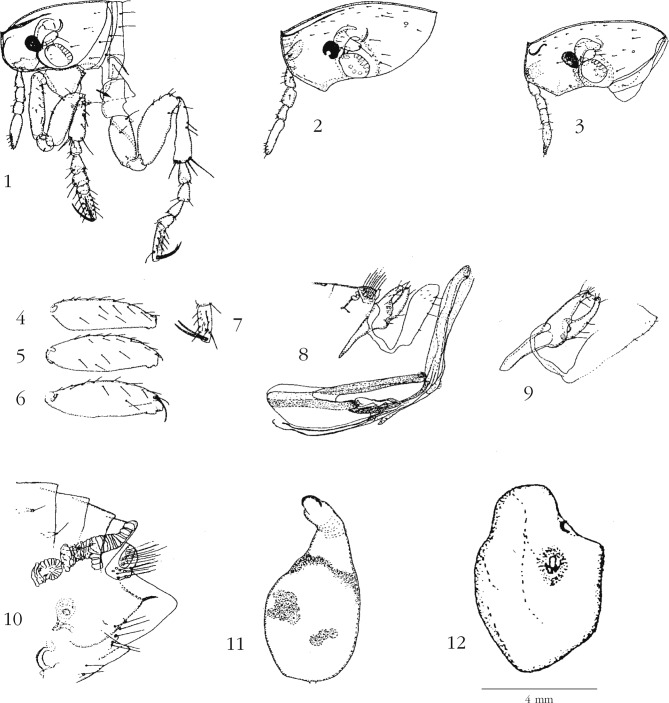
*Tunga bonneti* et *Tunga libis*. 1. *T. bonneti* n. sp., holotype, capsule céphalique, thorax et pattes I et II ; 2, 3. *T. bonneti*, capsule céphalique de deux femelles paratypes ; 4. *T. bonneti*, holotype, fémur III ; 5. *T. bonneti*, allotype, fémur III ; 6. *T. libis*, holotype, fémur III ; 7. *T. bonneti*, holotype, distitarsomère ; 8. *T. bonneti*, holotype, segment IX et phallosome ; 9. *T. libis*, holotype, segment IX ; 10. *T. bonneti*, femelle paratype, segments terminaux ; 11. *T. bonneti*, autre femelle paratype, spermathèque d’un néosome ; 12. *T. bonneti*, femelle paratype, néosome : la capsule céphalique est vue de face, le segment anal est situé plus haut et sur l’autre côté du néosome. Toutes ces figures sont à la même échelle, y inclus la spermathèque, sauf la figure 12.

Capsule céphalique (figures 1–3 à comparer à la figure 1 *in* Smit, 1962, *T. libis*, ♂ holotype) : elle est nettement convexe dans les deux sexes. OEil très pigmenté présentant, en fonction de la mise au point, une encoche ventrale ou une lacune médiane incolore. Front anguleux montrant la petite corne apicale classique du genre *Tunga*. Massue antennaire globuleuse avec six organes sensoriels (cf. Rothschild *et al.*, 1986) ; sur le bord antérieur du pédicelle on note une soie, sur le bord postérieur du pédicelle une soie chez le mâle, une ou deux chez les femelles. Palpe maxillaire de quatre articles dont l’apical est au moins aussi long que les articles II + III ; il atteint le trochanter chez le mâle, le dépasse plus ou moins chez la femelle. Stipe entièrement inclus dans la capsule céphalique ne montrant pas de denticulation. Palpe labial classiquement de deux articles ; comme les laciniae et l’épipharynx, il dépasse de peu le trochanter chez le mâle et il le dépasse très nettement chez la femelle. Rares soies, d’ailleurs de très faible taille, sur la partie occipitale, chez le mâle, nombreuses chez la femelle. Un léger processus génal est visible chez les femelles.

Thorax (figure 1) : ainsi que Smit (1962) l’a noté à propos de *T. libis* comme étant typique du groupe *caecata*, la séparation des trois segments thoraciques (et du tergite I) sont décelables dorsalement, leur fusion n’étant pas totale. Deux soies, moyennes, sont visibles en zone médiane sur les pro-, méso- et métathorax. Stigmate du métépiméron arrondi, petit ; deux soies fines, seulement, sont visibles sur ce segment. Patte I (figure 1) : *procoxa* plus ou moins triangulaire avec une micro-soie sur le bord ventral ; huit à neuf petites soies visibles sur la *coxa* ; trochanter arrondi avec une seule micro soie ; quatre soies moyennes sur le bord dorsal du fémur, une seule sur le bord ventral ; tibia montrant une soie épaissie sur son bord distal et quatre plus petites ; deux soies épaissies à sa base, l’une antérieure, l’autre postérieure ; les trois premiers segments du tarse sont aussi longs, ensemble, que le tibia ; les deux premiers segments sont légèrement plus longs que larges ; les deux derniers, distitarsomère exclus, aussi longs que larges ; distitarsomère trois à quatre fois plus long que large, portant quatre soies latérales des deux côtés, et environ une dizaine de micro-soies plantaires ; griffes un peu plus courtes que ce segment, doucement courbées, pratiquement sans allex. Patte II (figure 1, la flèche montre le glissement, arbitraire, vers la droite que nous avons donné au dessin de la *mesocoxa* pour éviter des superpositions d’images) : *mesocoxa* rectangulaire ; *coxa* allongée montrant un renforcement transversal : deux soies seulement sont notées (une médiane, une basale) ; fémur portant diverses soies à sa base ; tibia allongé, un peu moins long que le fémur, montrant trois soies sur son bord distal et quatre à son apex : deux relativement longues, une moyenne et une courte ; les quatre premiers segments tarsaux sont pratiquement de même longueur ; distitarsomère montrant quatre soies latérales dont la dernière est courte. Patte III (figures 4, 5, 7) : *metacoxa* triangulaire ; *coxa* courbe, avec un renforcement vertical, une forte sclérification “en crochet” à son bord antérieur, une soie moyenne en avant de ce crochet, une autre un peu plus développée en arrière et enfin sept à huit soies petites et fines à distance du bord antérieur ; trochanter courbé, deux fois plus long que large ; fémur (figures 4, 5) long portant huit soies marginales et des rangées latérales de petites soies fines ; la grande soie apicale externe, dite “de protection”, est manquante (comparer avec la figure 6 montrant le fémur de l’holotype de *T. libis*) ; tibia étroit à sa base, s’élargissant vers son apex, portant sur son bord postérieur trois encoches munies de deux soies fortes, une située à la base, une au milieu, une à l’apex ; le bord antérieur (ou ventral) ne montre que les soies apicales, deux longues et deux courtes, la plus longue dépassant nettement l’apex du premier tarsomère ; les quatre premiers tarsomères sont de longueur décroissante, le segment I étant presque aussi long que les segments II + III ; le segment II porte une très longue soie courbe dépassant légèrement l’apex du distitarsomère ; celui-ci montre quatre soies latérales d’un côté, trois de l’autre (figure 7). Chez les femelles néosomiques “âgées”, ne subsistent en général que *coxae* et trochanters, comme il est de règle.

Abdomen du mâle : les six premiers tergites montrent le même type de stigmate que le métépiméron, circulaire et petit ; le tergite VII a un stigmate en L inversé, classique. La sétation de tous ces segments est de une petite soie insérée au-dessus du stigmate ; entre stigmate et soie, on note une ou deux placoïdes pour les segments I à VI. *Sensilium* montrant huit fossettes. Segment IX (ou génital) (figure 8 à comparer à notre figure 9 : holotype de *T. libis*) : manubrium court, sa longueur est voisine de celle du basimère ; ce dernier est allongé, de morphologie évoquant, par exemple, celle du genre *Leptopsylla* (Ceratophyllidae – Leptopsyllinae), ressemblance évidemment fortuite ; il porte cinq ou six soies latérales et trois ou quatre apicales, puis marginales ; une soie basale et une latéroventrale ; telomère doucement arqué avec quatre soies plus ou moins apicales et une soie inféro-marginale ; sa marge antérieure est masquée par le bord postérieur du basimère ; sternite IX à bras proximal sinueux, à bras distal large à l’apex qui est fortement oblique en bas et en arrière ; il est peu sclérotisé, portant de rares soies marginales. Phallosome (figure 8 et photographie 1) de morphologie classique pour le genre, montrant une articulation médiane typique du genre *Tunga* ; la partie antérieure (pré-articulaire) est un peu plus longue que la partie distale. Sur la photographie, la forme canaliculaire du phallosome est bien visible.

**Photographie 1.**
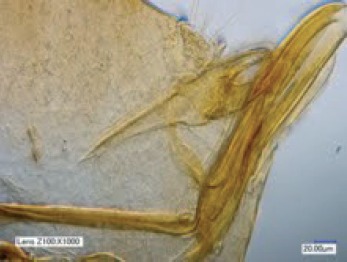
*Tunga bonneti*, holotype, phallosome : photographie effectuée en grande profondeur de champ avec le microscope numérique VHX-1000 (Keyence, France).

**Photographie 2.**
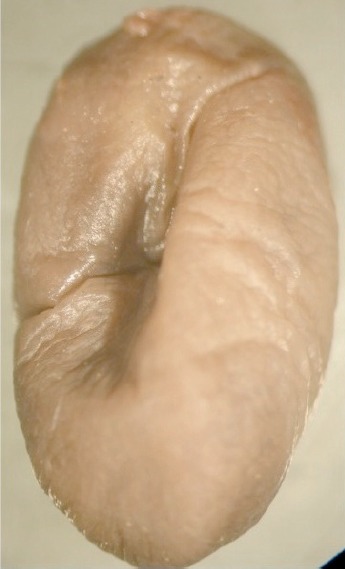
Néosome de *Tunga bonneti* extrait de la queue chez *Phyllotis darwini*, la capsule céphalique est montrée en position antérieure.

**Photographie 3.**
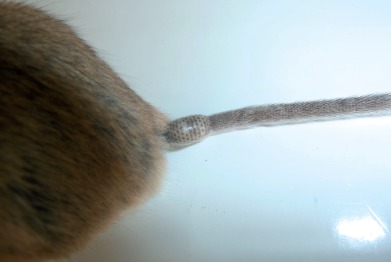
Néosome de *Tunga bonneti* situé, comme classiquement chez cette espèce, à la base de la queue en situation dorsale ; de plus, le segment anal du parasite est lui aussi dorsal.

Abdomen de la femelle (figure 10) : chez la femelle non fixée, les stigmates du métépiméron et des tergites II à IV sont petits et ronds ; les suivants sont grands et “floriformes” ; le tergite VIII montre un apex oval avec une plage d’environ six soies à sa base ventrale. Sternite VIII avec une projection apicale en “griffe” sclérotisée et doucement crénelée ; trois soies sont insérées au-dessus de celle-ci. Le *ductus bursae* (ou *bursa copulatrix*) est net ; en plus de ce conduit, seule l’*area cribriformis* (45 μm de diamètre) et le départ du *ductus spermathecae* sont visibles. Chez les femelles néosomiques, la sétation est indiscernable ; les stigmates V à VIII sont classiquement hypertrophiés et regroupés à l’extrémité de l’insecte. Le néosome est allongé (photographies 2, 3, 5), en “ballon de rugby”, mais souvent très déformé (figure 12) au niveau de la queue ; on peut, peut-être, regarder comme expansions du néosome des bourrelets verticaux, assez souvent présents mais irréguliers, encadrant et protégeant la capsule céphalique ; l’abdomen est distendu dorso-ventralement. Vers l’avant (intérieur de l’organe parasité), il recouvre thorax et capsule céphalique ; vers l’arrière, tout le segment anal (*sensilium*, orifices des trachées, anus et orifice de ponte) est projeté à l’extérieur où la saillie qu’il forme permet d’identifier, à l’oeil nu, le genre-parasite en cause. La plus grande dimension de ce néosome est parallèle au grand axe de la queue de l’hôte, ou moins fréquemment, du pavillon de l’oreille ; il est alors plus arrondi (photographie 4). Capsule céphalique et segment anal ne sont jamais diamétralement opposés, l’un pouvant être plus haut, ou plus bas situé, que l’autre (*cf*. figure 12). Le néosome mesure environ 6 mm de hauteur sur 4 mm de largeur ; les dimensions extrêmes allant de 10 × 5 mm à 5,5 × 2,5 mm. La spermathèque (figure 11) est grande, plus ou moins subsphérique et très pigmentée au niveau de la *bulga* ; la *hilla* est courte, légèrement arquée, incolore, son apex est sclérotisé et évoque une *papilla*. La cuticule de la *bulga* montre des motifs jointifs polygonaux ; son diamètre, ou sa largeur, est de 230 μm, ce qui correspond pratiquement à sa longueur ; longueur de la *hilla* 132 μm ; la longueur totale de la spermathèque (*bulga* + *hilla*) est de 390 μm. Cette spermathèque est pratiquement deux fois plus volumineuse que celle de *Tunga penetrans*, pour des insectes de taille identique, et est de forme très différente.

**Photographie 4.**
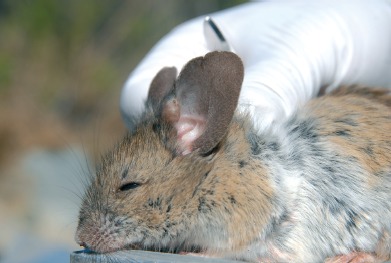
Néosome de *Tunga bonneti*, *in situ* au niveau du pavillon de l’oreille, même espèce-hôte.

**Photographie 5.**
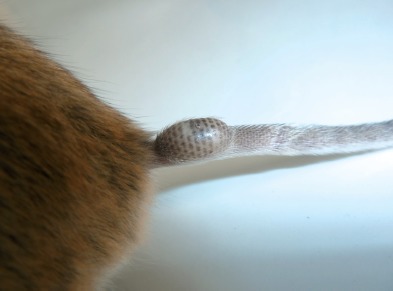
Néosome lésionnel de *T. bonneti* au niveau de la queue ; noter l’aplatissement de la queue en aval du parasite.

**Photographie 6.**
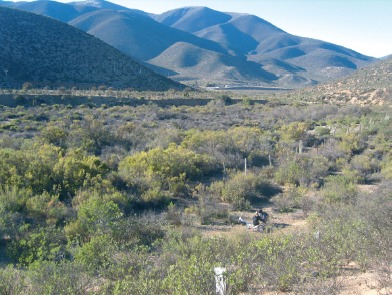
Biotope, Parque Nacional Las Chinchillas (31° 30’ 34’’ S, alt. 546 m).

**Photographies 7, 8.**
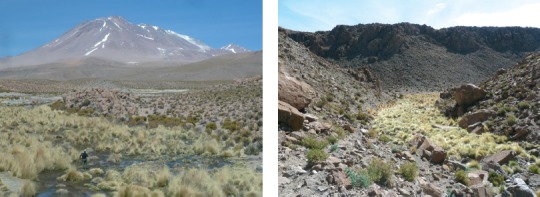
Biotope, La Quebrada del Inca (21° 10’ 03’’ S, alt. 3 794 m) ; on voit au fond de la photographie 7 le volcan Aucanquilcha.

Dimensions : mâle holotype : 0,9 mm ; femelles libres : 0,8 à 0,9 mm (allotype : 0,9), néosomes 6 à 10 mm dans la plus grande dimension, en l’occurrence, la hauteur.

### Discussion

Selon les critères retenus par Smit (1962) pour *T. libis*, notre espèce qui en est voisine appartient au groupe *caecata*. C’est pour cet auteur le groupe le plus primitif des deux (l’autre étant le groupe *penetrans*), basé sur :

- les hôtes qui sont ici des rongeurs Muridés placés soit, dans les Murinés (pour *caecata*, *caecigena*, *callida*), soit, le plus souvent dans les Sigmodontinae (pour *caecata* – qui semble donc moins spécifique que les autres –, *libis*, *monositus*, *bossii*, *bonneti*), suivant les espèces ;
- le fait que le pronotum ne soit pas totalement fusionné avec le mésonotum ;
- et que le distitarsomère de la patte III porte trois ou quatre paires de soies latérales, au lieu de deux. Barnes & Radovsky (1969) en notent huit à dix chez *T. monositus*, ce qui conforte finalement la scission effectuée par Smit

La création par Wang (1976) du sous-genre *Brevidigita* pour les deux *Tunga* chinoises, *caecigena* et *callida*, espèces auxquelles Lewis (2009) a ajouté *caecata*, mais non *libis*, ne nous paraît pas justifiée, même si l’on ne considère que les taxa chinois. Cette opinion est également celle de Linardi & Guimarães (2000).

Les femelles enkystées de *T. bonneti* n. sp. sont immédiatement séparables de toutes les autres femelles connues dans le genre *Tunga*, par la forme du néosome qui est nettement ovalisé (plus ou moins en ballon de rugby) dans la localisation la plus fréquente, la queue, et ne montre aucune réelle protubérance ou lobe. Rappelons que cette “ovalisation” s’effectue de haut en bas et non d’avant en arrière, c’est-à-dire que portion céphalique et portion anale sont disposées de part et d’autre du “ballon” et non aux deux pôles.

Mâles et femelles se caractérisent dans les deux sexes par la présence d’un oeil grand et très pigmenté ce qui exclus *caecata*, *travassosi*, *caecigena*, *bondari* (chez ce dernier, l’oeil est pigmenté mais petit), *monositus*, *callida* et *bossii*. Pour les espèces restantes, ayant donc un oeil grand et pigmenté, la soie apicale externe dite “de protection” de l’extrémité apicale du fémur manque chez *bonneti* ; chez *penetrans* et *trimamillata*, elle existe, bien que courte et trapue ; elle est grande chez *libis*. Nous ne savons rien de son existence, ou non, chez *terasma*, mais cette espèce sera facilement écartée de *T. bonneti* par la taille du segment IV du palpe maxillaire : il est, chez *terasma*, nettement plus court que les segments II + III, alors que chez *bonneti*, ce segment est au moins aussi long que ces segments.

## Spécificité

Les hôtes des *Tunga* liées aux rongeurs sont généralement très ciblés. Pour *T. bonneti*, seuls deux rongeurs Sigmodontinae sont notés, *Phyllotis darwini* et *Ph. xanthopygus*. Divers autres petits mammifères ont été collectés dans les stations répertoriées ci-dessous ou en d’autres points du Chili, mais tous se sont révélés négatifs. Citons *Thylamis elegans*, *Th. pallidor*, *Rattus norvegicus*, *Octodon degus*, *O. lunatus*, *Akodon olivaceus*, *Abrothrix longipilis* et *A. berlepschii*. Les *Thylamis* sont des Didelphidae ; tous les autres sont des rongeurs Sigmodontinae, sauf *R. norvegicus* qui est un Murinae.

## Chorologie

### Description Des Stations De Collecte

Celles-ci, pour le moment, s’étagent pratiquement de 33° 30’ à 21° 10’ de latitude sud. Au Chili, Macchiavello (1948) signale *T. penetrans* de Chiloë à Iquique, soit de 43° à 20° 13’, ce qui repousse vers le sud la répartition connue des *Tunga* dans ce pays. Il est vrai que cet auteur mentionne, en dehors de l’homme, de nombreux hôtes (ici, il s’agit du porc) dont certains, comme les rats, sont étonnants pour *T. penetrans*. Nous n’avons pas pu savoir ce qu’était devenu ce matériel et nous ne pouvons écarter l’hypothèse que certaines de ces puces appartiennent à un autre taxon que *penetrans*.

### Répartition De *T. bonneti* Au Chili (carte 1)

A – Dunas Las Cruces, province de Santiago. Dunes secondaires avec une végétation assez dense, à 1 km de la côte, près de Las Cruces, à environ 50 km au sud de Valparaiso (Smit, 1968) ; coordonnées : 33° 29’ 50’’ S – 71° 37’ 22’’ O. L’altitude est manifestement voisine de 0 m.

**Carte 1.**
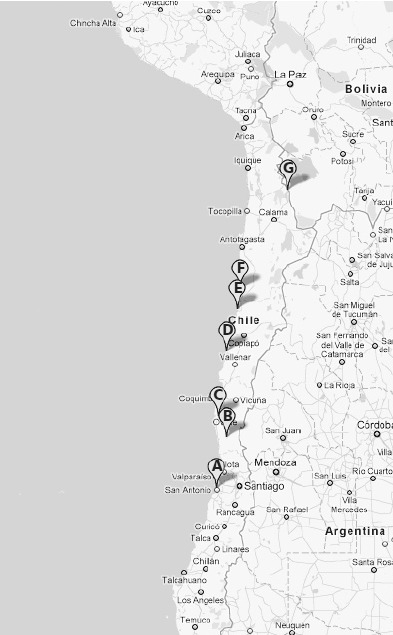
Répartition de *Tunga bonneti*, toutes les stations actuellement connues sont au Chili. A. Dunas de las Cruces (Santiago), 33° 29’ 50’’ S – 71° 37’ 22’’ O, altitude voisine de 0 m ; B. Reserva Nacional Las Chinchillas (Choapa), 31° 30’ 34’’S – 71° 06’ 23’’ O, 546 m ; C. Parque Nacional Bosques de Fray Jorge (Limari), 30° 38’ 25’’ S – 71° 31’ 09’’ O, 254 m ; D. Parque Nacional Llanos de Challe (Huasco), 28° 02’ 36’’ S – 71° 06’ 52’’ O, 104 m ; E. Parque Nacional Pan de Azúcar (Chañaral), 26° 09’ 35’’ S – 70° 40’ 01’’ O, 30 m ; F. Tal-Tal (Antofagasta), 25° 24’ 20’’ S – 70° 30’ 24’’ O, 25 m ; G. Quebrada del Inca (El Loa), près d’Ollagüe, 21° 10’ 03’’ S – 68° 19’ 54’’ O, 3 794 m. Cartographie Googlemaps 2011.

B et C – Reserva Nacional Las Chinchillas (photographie 6) (RNCH) (31° 30’ 34’’ S – 71° 06’ 23’’ O), altitude 546 m, province de Choapa et Parque Nacional Bosques de Fray Jorge (PNFJ) (30° 38’ 25’’ S – 71° 31’ 09’’ O), altitude 254 m, province de Limarí. Ces stations se situent dans une zone à climat méditerranéen aride caractérisé par des précipitations en automne et en hiver, avec une aridité accentuée pendant les saisons chaudes (Di Castri & Hajek, 1976). La végétation de la RNCH est composée d’arbustes xérophytiques, cactus, *Puya* spp., *Acacia caven* et d’arbres sclérophylles dans les vallées (Gajardo, 1994). Au PNFJ, la végétation est identique mais, en plus, les cimes des collines côtières ont des forêts hygrophiles composées principalement d’Olivillos (*Aextoxicon punctatum*) qui se maintiennent grâce au brouillard et aux nuages présents pendant la plus grande partie de la journée (Villagrán *et al.*, 2004).

D, E et F – Parque Nacional Llanos de Challe (PNLCH) (28° 02’ 36’’ S – 71° 06’ 52’’ O), altitude 104 m, province de Huasco, Parque Nacional Pan de Azúcar (PNPA) (26° 09’ 35’’ S – 70° 40’ 01’’ O), altitude 30 m, province de Chañaral, et Tal-Tal (25° 24’ 20’’ S – 70° 30’ 24’’ O) altitude 25 m, province de Antofagasta, sont localisés dans une région de climat méditerranéen per-aride, présentant une période de sécheresse au printemps et en été, et une pluviométrie concentrée sur les mois froids (Di Castri, 1968). Cependant, il n’y existe pas de régime des pluies bien défini (Di Castri & Hajek, 1976). La végétation se caractérise par la présence de petits arbustes xérophytiques, de cactus et de plusieurs espèces géophytes et éphémères qui fleurissent après des précipitations occasionnelles, donnant lieu au phénomène du “désert fleuri” (Gajardo, 1994).

G – Quebrada del Inca (photographies 7, 8) (21° 10’ 03’’ S – 68° 19’ 54’’ O), altitude 3 794 m, près d’Ollagüe, province de El Loa, est située dans une région au climat tropical d’altitude (Di Castri, 1968). Cette zone se caractérise par la présence de massifs de haute altitude qui provoquent des précipitations régulières pendant toute la saison chaude, phénomène connu comme “hiver bolivien” (Di Castri & Hajek, 1976). Les communautés végétales sont déterminées par le relief et par la présence de cours d’eau ; elles sont représentées, principalement, par des herbes de la famille Juncaceae, *Azorella compacta* et des associations de *Polylepis* spp. (Gajardo, 1994).

### Analyse

Si l’on considère les altitudes, allant dans ces relevés du niveau de la mer à près de 3 800 m, il nous semble évident que ce facteur ne peut intervenir dans la répartition de *T. bonneti*. En revanche, l’examen des biotopes (photographie 6 : Reserva Nacional las Chinchillas et photographies 7, 8 : Quebrada del Inca) nous montre de façon évidente, le “pourquoi” de cette répartition. Il s’agit de biotopes “ouverts” et donc à végétation basse, à sol montrant une fine granulométrie. Cette notion de granulométrie était déjà connue pour *T. penetrans* (*cf*. *inter alia* Bonnet, 1867 ; Manson, 1903 ; Doucet, 1969 ; Degeilh & Beaucournu, 2003…), d’où les noms de “*sandflea*” des Anglo- Saxons ou de “*sandfloh*” des germanophones. Les brèves descriptions données par Barnes & Radovsky (1969) ou par Hastriter (1997) des biotopes de collecte de *T. monositus* semblent aller dans le même sens. Il s’agit peut-être d’une coïncidence, mais il est fréquent de trouver dans un gîte donné et sur la même espèce-hôte, *T. bonneti* et *Hectopsylla* spp., en général *H. cypha* Jordan, 1942. Ces deux genres sont, traditionnellement, considérés comme proches, mais cette position taxonomique sera ultérieurement discutée.

## Dermecos

Le terme “dermecos”, que nous estimons devoir utiliser par la précision qu’il apporte, a été créé par Smit (1972) pour désigner “*the microhabitat created by the host-skin and its outgrowths*”. Actuellement, les sept espèces de Tungidae parasitant des rongeurs dont la description est parue (*caecata*, *caecigena*, *libis*, *monositus*, *callida*, *bossii* et *bonneti*) ont, à l’inverse de ce qui est observé chez *T. penetrans*, par exemple, une localisation précise, un dermecos précis, sur leur hôte.

En général, un seul dermecos concerne une *Tunga* donnée dans le groupe “caecata”. Toutefois, chez *T. bonneti* deux localisations sont connues, la base de la queue, et plus rarement le pavillon de l’oreille :

- La queue (photographies 2, 3). Cette localisation n’avait été signalée qu’une fois, théoriquement à propos de *T. caecigena*, par Yang (1955, cité et commenté par Smit, 1962, in Jordan, 1962) : “*Once it was also found at the base of the tail of a specimen of* Rattus rattus”, mais Smit précise “*may refer to the closely related* Tunga callida *Li* & *Chin, 1957 (from Yunnan), the females of which bury themselves at the rear end of the host, especially around the anus*”. Pour *T. bonneti* la localisation caudale est extrêmement fréquente, puisque nous l’avons noté 60 fois sur 68 néosomes ! Toutes ces puces ne furent malheureusement pas conservées en alcool ; toutefois, un certain nombre d’entre elles furent photographiées et toutes furent comptabilisées (la plupart d’ailleurs comme larves de Cuterebridae). C’est près de la base de la queue, à un centimètre environ, que le néosome se situe ; la plupart du temps, à en juger par les photographies disponibles, le segment anal est en situation dorsale. Nous devons noter que cette “tumeur” caudale a dû, bien souvent, être négligée car considérée comme un cal cicatriciel résultant d’une blessure ou d’une morsure. Nous présentons une photographie (5) montrant le seul cas observé où une lésion résulte de cette implantation. Chez *T. bossii*, la base de la queue est parasitée, mais à sa face inférieure, au niveau de l’anus et des organes génitaux.
- Le pavillon de l’oreille (photographie 4), autre dermecos connu pour *T. bonneti*, a été trouvé parasité sept fois (sur un total de 68). Dans le seul cas étudié, la pénétration de la puce s’est effectuée par la face externe du pavillon de l’oreille ; en étudiant les autres photographies, il ne semble pas en être de même dans la plupart des autres cas, où tantôt la face interne, tantôt le bord libre de l’oreille, comme chez *Dermatophilus lagrangei* (= *T. caecigena*) (*in* Roubaud,1925) semblent concernés.

## Phénologie

Quatre exemplaires libres, c’est-à-dire venant de subir la nymphose imaginale, sont connus à ce jour chez *T. bonneti* : deux femelles collectées entre le 13 octobre et le 25 novembre (Smit, 1968), un mâle et une femelle vers le 15 décembre. Si l’on se fie à des chiffres aussi bas (mais il est assez exceptionnels pour le genre *Tunga* d’avoir à la fois “autant” de spécimens libres !) ; on a l’impression que *T. bonneti* est une espèce univoltine. Smit (*in* Jordan, 1962) formulait la même possibilité pour *T. caecigena*, l’une des deux espèces chinoises.

Les relevés de piégeage effectués au Chili nous montrent nettement la phénologie de *T. bonneti*. Si l’on considère l’ensemble des stations ayant livré cette espèce, on note que cette espèce n’est pas collectée pendant les mois de avril, mai et juin (soit l’automne austral) ; son abondance apparaît brusquement de juillet à décembre (avec 46 néosomes), puis elle montre une assez brusque décroissance de janvier à mars (22 néosomes) pour disparaître ensuite. Compte tenu de la latitude, c’est donc essentiellement une puce de saison froide. Cette phénologie est en accord avec les données publiées pour d’autres *Tunga*, par exemple *monositus* (Hastriter, 1997), ou *caecigena* qui vit en région paléarctique orientale (Chine) et est considérée comme espèce hivernale (Roubaud, 1925 ; Smit *in* Jordan, 1962).

## Conclusions

L’étude minutieuse de nos relevés de piégeage, nous a montré quels étaient les hôtes bien sûr, mais aussi et surtout les biotopes et les périodes de l’année où nous pouvions espérer collecter cette puce. En tenant compte de la latitude, beaucoup plus que de l’altitude, nous pensons que nos données sont applicables à d’autres *Tunga* et peuvent, en particulier aider à la capture des mâles rares ou inconnus dans de nombreux cas.

## References

[R1] Avelar D.M. & Linardi P.M. Use of multiple displacement amplification as pre-polymerase chain reaction (Pre-PCR) to amplify genomic DNA of siphonapterids preserved for long periods in scientific collections. Parasites & Vectors, 2010, 86, 1–6. 10.1186/1756-3305-3-86 PMC2945329 20840790

[R2] de Avelar D.M. de, Linharès A.X. & Linardi P.M. A new species of *Tunga* (Siphonaptera: Tungidae) from Brazil with a key to the adult species and neosomes. Journal of Medical Entomology, 2012, 49, 23–28. 22308767 10.1603/me11111

[R3] Barnes A.M. & Radovsky F.J. A new *Tunga* (Siphonaptera) from the nearctic region with description of all stages. Journal of Medical Entomology, 1969, 6, 19–36. 5775471 10.1093/jmedent/6.1.19

[R4] Beaucournu J.C. & Gallardo M.H. Catalogue provisoire des Puces du Chili (Insecta : Siphonaptera) (2^ème^ partie). Bulletin de la Société Française de Parasitologie, 1992, 10, 93–130.

[R5] Bonnet G. Mémoire sur la Puce pénétrante ou Chique. J.-B. Baillière et Fils, Paris, 1867, 102 p., 2 pl.

[R6] Degeilh B. & Beaucournu J.C. Tungose, *in*: Parasitoses et Mycoses courantes de la peau et des phanères (chapitre 5, pp 55–63 ; figs 5–1 à 5–15). Elsevier SAS, 2003.

[R7] Di Castri F. Esquisse écologique du Chili, *in*: Biologie de l’Amérique australe. Centre National de la Recherche Scientifique, Paris, 1968, 4, 7–52.

[R8] Di Castri F. & Hajek E. Bioclimatologia de Chile. Vicerectoria Academica, Universidad Católica de Chile. Edición de la Universidad Católica de Chile, 1976, 225 p.

[R9] Doucet J. Tungose. Encyclopédie Médico-Chirurgicale, Maladies Infectieuses, Paris, 1969, 8120 D10, 4 p.

[R10] Gajardo R. La vegetación natural de Chile: classificación y distribución geográfica. Santiago, Editorial Universitaria, 1994, 165 p.

[R11] Hastriter M.W. Establishment of the tungid flea, *Tunga monositus* (Siphonaptera: Pulicidae), in the United States. Great Basin Naturalist, 1997, 57, 281–282.

[R12] Jordan K. Notes on *Tunga caecigena* (Siphonaptera: Tungidae). Bulletin of the British Museum (Natural History), Entomology, 1962, 12, 353–364.

[R13] Lewis R.E. Siphonaptera. Part I. Supraspecific classification. Part II. Alphabetical genus and species list. Part III. Alphabetical species/subspecies list, 16th edition Lewis R.E. (ed.), 2009, 61 p.

[R14] Linardi P.M. & Botelho J.R. Algumas observaçoes sobre Tunga do grupo “caecata” parasitando roedores silvestres. Resumos IX Congres. Soc. Bras. Parasit., Fortaleza, 1985, p. 162.

[R15] Linardi P.M. & Guimarães L.R. Sifonápteros do Brasil. Museu de Zoologia – USP, FAPESP São Paulo, Brazil, 2000, 291 p., 365 figs.

[R16] Macchiavello A. Siphonaptera de la costa sur-occidental de America (Primera lista y distribución zoo-geografica). Boletín de la Oficina Sanitaria Panamericana, 1948, 27, 412–460.

[R17] Manson P. Tropical diseases. A manual of the diseases of warm climates. Classell and Company, Ltd, London, Paris, New York & Melbourne, 1903, 756 p.

[R18] Roubaud E. Une nouvelle espèce de puce-chique pénétrante, parasite des rats en Chine : *Dermatophilus lagrangei* n. sp. Bulletin de la Société de Pathologie exotique, 1925, 18, 399–405.

[R19] Rothschild M. & Schlein Y. & Ito S.A. A colour atlas of insect tissues, via the flea. Wolfe Science Book, London, 1986, 184 p.

[R20] Smit F.G.A.M. A New sand-flea from Ecuador. The Entomologist, 1962, 95, 89–93.

[R21] Smit F.G.A.M. Siphonaptera taken from formalin-traps in Chile. Zoologisches Anzeiger, 1968, 180, 220–228.

[R22] Smit F.G.A.M. On some adaptative structures in Siphonaptera. Folia Parasitologica, Praha, 1972, 19, 5–17. 4670804

[R23] Villagrán C., Armesto J.J., Hinojosa L.F., Cuvertino J., Pérez C. & Medina C. El enigmático origen del bosque relicto de Fray Jorge, *in*: Historia natural del Parque Nacional Bosque de Fray Jorge (pp. 3–43). Ediciones Universidad de la Serena, Chile, 2004, 318 p.

[R24] Wang Dwen-Ching. The chinese *Tunga* (Siphonaptera: Tungidae). Acta Entomologica Sinica, 1976, 19, 117–118 (en chinois, seul le titre est traduit en anglais).

[R25] Whiting M.F., Whiting A.S., Hastriter M.W. & Dittmar K. A molecular phylogeny of fleas (Insecta: Siphonaptera): origins and host associations. Cladistics, 2008, 24, 1–31.

